# Work-Related and Personal Factors Associated With Mental Well-Being During the COVID-19 Response: Survey of Health Care and Other Workers

**DOI:** 10.2196/21366

**Published:** 2020-08-25

**Authors:** Bradley A Evanoff, Jaime R Strickland, Ann Marie Dale, Lisa Hayibor, Emily Page, Jennifer G Duncan, Thomas Kannampallil, Diana L Gray

**Affiliations:** 1 Washington University School of Medicine St. Louis, MO United States; 2 Healthier Workforce Center of the Midwest University of Iowa Iowa City, IA United States; 3 Human Resources Washington University in St. Louis St. Louis, MO United States

**Keywords:** COVID-19, coronavirus, pandemic, mental health, health care workers, remote work, worker well-being, occupational health

## Abstract

**Background:**

The response to the severe acute respiratory syndrome coronavirus 2 (SARS-CoV-2) pandemic has created an unprecedented disruption in work conditions. This study describes the mental health and well-being of workers both with and without clinical exposure to patients with coronavirus disease (COVID-19).

**Objective:**

The aim of this study is to measure the prevalence of stress, anxiety, depression, work exhaustion, burnout, and decreased well-being among faculty and staff at a university and academic medical center during the SARS-CoV-2 pandemic and describe work-related and personal factors associated with their mental health and well-being.

**Methods:**

All faculty, staff, and postdoctoral fellows of a university, including its medical school, were invited in April 2020 to complete an online questionnaire measuring stress, anxiety, depression, work exhaustion, burnout, and decreased well-being. We examined associations between these outcomes and factors including work in high-risk clinical settings and family/home stressors.

**Results:**

There were 5550 respondents (overall response rate of 34.3%). Overall, 34% of faculty and 14% of staff (n=915) were providing clinical care, while 61% of faculty and 77% of staff were working from home. Among all workers, anxiety (prevalence ratio 1.37, 95% CI 1.09-1.73), depression (prevalence ratio 1.28, 95% CI 1.03-1.59), and high work exhaustion (prevalence ratio 1.24, 95% CI 1.13-1.36) were independently associated with community or clinical exposure to COVID-19. Poor family-supportive behaviors by supervisors were also associated with these outcomes (prevalence ratio 1.40, 95% CI 1.21-1.62; prevalence ratio 1.69, 95% CI 1.48-1.92; and prevalence ratio 1.54, 95% CI 1.44-1.64, respectively). Age <40 years and a greater number of family/home stressors were also associated with these poorer outcomes. Among the subset of clinicians, caring for patients with COVID-19 and working in high-risk clinical settings were additional risk factors.

**Conclusions:**

Our findings suggest that the pandemic has had negative effects on the mental health and well-being of both clinical and nonclinical employees. Mitigating exposure to COVID-19 and increasing supervisor support are modifiable risk factors that may protect mental health and well-being for all workers.

## Introduction

The severe acute respiratory syndrome coronavirus 2 (SARS-CoV-2) pandemic has created unprecedented disruption in social interactions and working conditions. Recent studies have described the effects of the pandemic on the mental health and well-being of frontline health care workers (HCWs) [1,2], and potential interventions to protect them [3-5]. Although concern over health and well-being has primarily focused on frontline HCWs, the pandemic has also affected working conditions in most other industries. Social and employment changes have led to concerns about an impending “second pandemic” of short- and long-term mental health issues [6], and predictions of a preventable surge of avoidable deaths from alcohol, drug use, and suicide [7]. Few studies describe the effects of the pandemic on the mental health and well-being of workers outside of health care. Such evidence is important for developing appropriate responses to the pandemic to preserve health and plan for economic and social recovery.

We describe results from the EMPOWER study (EMPlOyee Well-Being during Epidemic Response), which measured mental health and well-being among a large and diverse academic workforce, including those with and without clinical exposure to patients with coronavirus disease (COVID-19). The goals of the study were to measure the prevalence of stress, anxiety, depression, work exhaustion, burnout, and decreased mental well-being among faculty and staff at a university and its academic medical center during the SARS-CoV-2 pandemic; to compare mental health and well-being between clinical workers who were or were not caring for patients with COVID-19; and to identify other modifiable workplace and personal risk factors associated with mental health and well-being.

## Methods

### Study Design and Participants

We conducted a web-based survey of all benefits-eligible university employees (faculty, staff, and postdoctoral scholars) at Washington University in St. Louis, a private university with a large academic medical center where attending physicians and clinical staff are university employees. A separate survey was sent to physician trainees (residents and clinical fellows) and is not included in this report [8]. An email invitation was sent to all benefits-eligible employees on April 17, 2020, with a clickable link to a voluntary, anonymous online survey. A single reminder email was sent 10 days later. The survey period was approximately 4 to 5 weeks after the university had enacted work-from-home plans. The study was approved by the institutional review board of Washington University in St. Louis.

### Survey Instrument

The survey was designed to take less than 10 minutes to complete. Definitions and sources of personal factors, work factors and well-being variables used in the survey are shown in Multimedia Appendix 1. Demographic questions included age, race, household income; children, dependents, and other adults living at home; and work status of partner. Questions about work included current work status (on-site work involving clinical care, on-site work not involving clinical care, working from home, or not working). Those doing on-site work in clinical care were asked about the clinical setting, and if they had cared for patients with COVID-19. All participants were asked if they or a member of their household had received a medical diagnosis or positive test result for COVID-19 or if they had been exposed to someone with COVID-19.

The questionnaire also included three questions from the FSSB-SF [9], which measures supervisor behaviors supportive of family roles (eg, “Your supervisor makes you feel comfortable talking to him/her about your conflicts between work and nonwork”; “Your supervisor demonstrates effective behaviors in how to juggle work and nonwork issues”; “Your supervisor works effectively with employees to creatively solve conflicts between work and nonwork.”) We used the mean value of these three responses as the supervisor support variable. We also asked about 8 potential family/home stressors related to the pandemic (childcare, home schooling, caring for elderly relatives, having access to food and other necessities, being infected, friends and family being infected, keeping your job, and personal finances). These questions were asked in the format “Currently how stressed are you about…?” in a 5-point scale from “not at all” to “extremely” stressed. The number of stressors reported by each individual as “somewhat” to “extremely” were totaled to create a composite stress score (range 0-8).

### Outcome Measures

Study outcomes included stress, anxiety, and depression as measured by the Depression, Anxiety and Stress Scale - 21 Items (DASS-21) [10], burnout and work exhaustion as measured by the Professional Fulfillment Index (PFI) [11], and changes in well-being [12]. The DASS-21 is a validated instrument with scales that correlate well with other measures of depression, anxiety, and stress. Due to the PFI questionnaire structure, burnout was only assessed among HCWs. Self-reported changes in well-being comparing current to prepandemic status were assessed in five domains (overall, financial, physical, mental, and social) by the question “To what extent have COVID-19–related work/life changes impacted your well-being?” using a 4-point scale from “much worse” to “much better/somewhat better.”

### Statistical Analyses

We contrasted the proportions or means of outcomes between faculty and staff and those in different clinical settings. We then conducted univariable and multivariable Poisson regression with robust sandwich estimators to examine personal and work factors associated with six mental health and well-being outcomes described above: stress, anxiety, depression, burnout, work exhaustion, and changes in well-being.

In conducting these analyses, we selected 10 a priori potential personal and work factors as independent variables for multivariable analysis (supervisor support, clinical work, staff [versus faculty or postdoctoral fellow], exposure to people [or patients for clinicians] with a diagnosis of COVID-19, age, sex, race, annual household income, children aged under 18 years living at home, and composite stressor count). Results were expressed as prevalence ratios (PRs) with 95% CIs. Independent variables were dichotomized at the median scores or at relevant cut-points for ordinal variables. We categorized race and ethnicity as “underrepresented groups” (those identifying as Black/African American, Native American, Hawaiian/Pacific Islander, or Hispanic) and “Other.” The significance level was set at .05 and hypothesis tests were two-sided. All analyses were performed with R statistical software (Version 4.0.0; R Foundation for Statistical Computing) [13] and R studio (Version 1.2.504) [14].

### Patient and Public Involvement

The survey was developed in collaboration with the university human resources department and the employee wellness director to ensure sensitivity to current issues and to address emerging concerns about employee wellness during the pandemic response. Initial survey results have been shared with university leaders to highlight the mental health needs of employees. Study results are driving plans to communicate broadly with faculty, staff, and trainees to highlight mental health challenges faced by our workforce and to better publicize and encourage employees to utilize available mental health resources.

## Results

**Figure 1:**
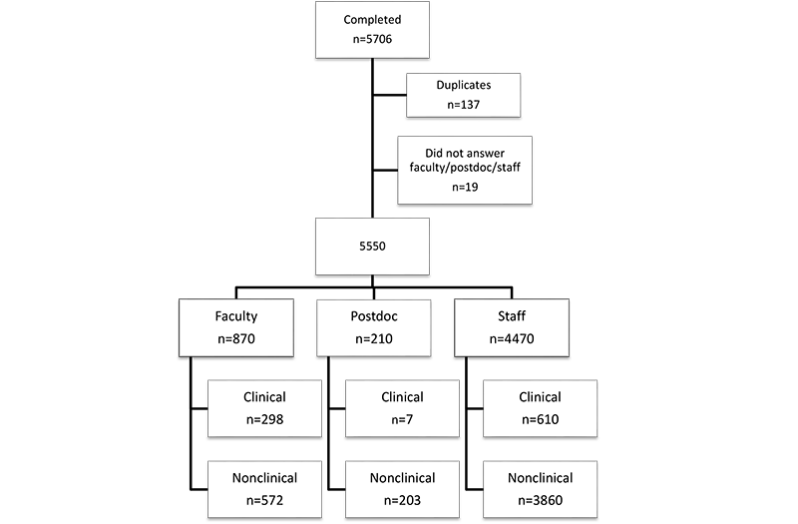
Survey response flowchart.

Email invitations were sent to all benefits-eligible university faculty, staff, and postdoctoral scholars (N=16,238). In total, 5706 responses were received (Figure 1); there were 5569 unique responses after the exclusion of 137 responses with a duplicate self-generated identifier that allows anonymous longitudinal follow-up. Of the remaining surveys, 19 were excluded for missing status as faculty, staff, or postdoctoral scholar, leaving 5550 surveys for analysis (870 faculty, 4470 staff, and 210 postdoctoral fellows). The overall response rate was 34.3% for unique surveys. Response rates were higher for staff than for faculty (40% versus 19.7%).

Table 1 compares demographics, work factors, and outcomes between faculty, staff, and postdoctoral fellows. Overall, 34.3% of faculty and 13.6% of staff reported working on-site in clinical operations while a majority of the faculty (60.6%) and staff (76.5%) were working from home. Smaller numbers worked on-site in nonclinical roles and few were not working. A majority of faculty (50.4%) reported that their workload increased after the COVID-19 workplace changes, as compared to 40.4% of staff and 21% of postdoctoral fellows. Overall, a majority of respondents reported being stressed (more than “a little bit”) about personal finances, keeping their jobs, and about themselves or friends or family becoming infected. Of those with children at home, a majority reported feeling stressed about homeschooling; most of those providing care to older relatives reported stress about their care. Distributions of most perceived stressors were significantly different across the faculty, staff, and postdoctoral fellows, with postdoctoral fellows more frequently reporting stress about childcare, homeschooling, and access to food and essential supplies. Faculty, staff, and postdoctoral fellows all reported a high prevalence of worsened overall well-being (58.3%) related to COVID-19 work or life changes. Moderate to high levels of stress were reported by 13%, anxiety by 13%, depression by 15.9%, and high work exhaustion by 43%.

**Table 1:** Comparison of demographics, personal factors, work factors, and outcomes between faculty, staff, and postdoctoral fellows^a^.

			Faculty (n=870)		Staff (n=4470)		Postdoctoral fellows (n=210)		Total (N=5550)		*P* value		
**Personal and family factors**													
	Age above 40 years, n (%)			624 (72.0)		2652 (59.5)		22 (10.5)		3298 (59.6)		<.001	
	**Gender, n (%)**												<.001
		Male	333 (38.4)		772 (17.3)		77 (37.0)		1182 (21.4)		N/A^b^		
		Female	523 (60.3)		3624 (81.3)		127 (61.1)		4274 (77.3)		N/A		
		Gender diverse	4 (0.5)		18 (0.4)		2 (1.0)		24 (0.4)		N/A		
		Prefer not to say	8 (0.9)		41 (0.9)		2 (1.0)		51 (0.9)		N/A		
	Underrepresented groups^c^, n (%)			68 (7.8)		482 (10.8)		26 (12.4)		576 (10.4)		.02	
	Annual household income <$70,000, n (%)			68 (8.2)		1551 (36.5)		133 (64.3)		1752 (33.2)		<.001	
	Living alone, n (%)			111 (12.8)		645 (14.5)		62 (29.8)		818 (14.8)		<.001	
	Two adults in health care with children, n (%)			68 (7.8)		58 (1.3)		2 (1.0)		128 (2.3)		<.001	
	Stressed about childcare^d^, n (%)			193 (46.5)		652 (36.7)		26 (53.1)		871 (38.9)		<.001	
	Stressed about home schooling^e^, n (%)			216 (61.7)		846 (56.8)		22 (84.6)		1084 (58.1)		.006	
	Stressed about relatives^f^, n (%)			87 (73.7)		560 (75.9)		12 (75.0)		659 (75.6)		.88	
	Stressed about essential supplies, n (%)			199 (23.0)		1341 (30.2)		77 (36.7)		1617 (29.3)		<.001	
	Stressed about being infected, n (%)			491 (56.5)		2556 (57.5)		101 (48.1)		3148 (57.0)		.03	
	Stressed about friends or family getting infected, n (%)			665 (76.7)		3347 (75.2)		130 (61.9)		4142 (75.0)		<.001	
	Stressed about keeping job, n (%)			288 (33.3)		2786 (62.6)		116 (55.5)		3190 (57.8)		<.001	
	Stressed about personal finances, n (%)			422 (49.2)		2698 (60.8)		110 (53.1)		3230 (58.7)		<.001	
	Number of stressors, mean (SD)			2.9 (1.9)		3.3 (1.9)		2.8 (1.8)		3.2 (1.9)		<.001	
	Any exposure to COVID-19, n (%)			142 (16.3)		272 (6.1)		11 (5.2)		425 (7.7)		<.001	
**Work factors**													
	**Current work, n (%)**												<.001
		Working onsite, clinical operations	298 (34.3)		610 (13.6)		7 (3.3)		915 (16.5)		N/A		
		Working onsite, nonclinical operations	33 (3.8)		339 (7.6)		18 (8.6)		390 (7.0)		N/A		
		Working at home	527 (60.6)		3421 (76.5)		183 (87.1)		4131 (74.4)		N/A		
		Not working	12 (1.4)		100 (2.2)		2 (1.0)		114 (2.1)		N/A		
	Supervisor support scale (range 1-5), mean (SD)			2.5 (1.0)		2.2 (1.1)		2.3 (1.1)		2.3 (1.1)		<.001	
	Increased workload since COVID-19 restrictions began, n (%)			426 (50.4)		1747 (40.4)		43 (21.0)		2216 (41.2)		<.001	
**Outcomes**													
	Worse overall well-being due to COVID-19–related work or life changes, n (%)			588 (67.8)		2490 (56.2)		130 (62.2)		3208 (58.3)		<.001	
	Worse financial well-being due to COVID-19–related work or life changes, n (%)			381 (43.9)		1291 (29.1)		60 (28.6)		1732 (31.4)		<.001	
	Worse physical well-being due to COVID-19–related work or life changes, n (%)			387 (44.6)		1938 (43.7)		88 (41.9)		2413 (43.8)		.77	
	Worse mental well-being due to COVID-19–related work or life changes, n (%)			604 (69.7)		3027 (68.1)		142 (67.6)		3773 (68.4)		.63	
	Worse social well-being due to COVID-19–related work or life changes, n (%)			703 (81.2)		3482 (78.5)		168 (80.4)		4353 (79.0)		.18	
	Mean well-being score, mean (SD)			2.3 (0.5)		2.4 (0.5)		2.4 (0.5)		2.4 (0.5)		<.001	
	Moderate to high depression (DASS), n (%)			133 (15.9)		676 (15.7)		39 (19.5)		848 (15.9)		.36	
	Moderate to high anxiety (DASS), n (%)			83 (10.0)		582 (13.5)		30 (14.9)		695 (13.0)		.02	
	Moderate to high stress (DASS), n (%)			105 (12.6)		552 (12.7)		39 (20.0)		696 (13.0)		.01	
	High work exhaustion, n (%)			419 (49.7)		1783 (41.3)		105 (51.2)		2307 (43.0)		<.001	

^a^Missing values for each variable (range 0%-4.8%) were omitted from percentage calculations. Percentages may not total 100 due to rounding. Categorical variables are displayed as n (%), while continuous variables are displayed as mean (SD). The chi-square test was used for categorical variables, while analysis of variance was used for continuous variables.

^b^N/A: not applicable.

^c^Underrepresented groups were those identifying as Black/African American, Native American, Hawaiian/Pacific Islander, or Hispanic.

^d^Percentages are among those with children only.

^e^Percentages are among those with children above preschool age only.

^f^Percentages are among those with elderly parents or relatives only.

Multivariable analyses of associations between these outcomes and a common set of work and personal factors among all respondents showed that three factors were statistically significantly associated with a higher prevalence of all five outcomes (Table 2, univariable analyses in Multimedia Appendix 2): poor supervisor support, a higher number of family/home stressors, and age <40 years. Working on-site in clinical operations was associated with higher anxiety and lower mean well-being; being a staff member (rather than faculty or postdoctoral fellow) was associated with better well-being and lower prevalence of stress and work exhaustion. Reported exposure to COVID-19 (diagnosis in self or family, or exposure to someone likely to have COVID-19) was associated with higher stress, anxiety, depression, and work exhaustion. A household income of <$70,000 was associated with a higher prevalence of stress, anxiety, and depression. Women were more likely to report experiencing anxiety, work exhaustion, and decreased well-being. Unanticipated protective factors were also notable: having children at home was associated with a lower prevalence of anxiety and depression, and underrepresented racial/ethnic groups were less likely to report stress, depression, or decreased well-being.

**Table 2:** Multivariate associations between personal factors, work factors, and well-being among all participants (N=5550).

Variable	Moderate to high stress (DASS), PR (95% CI)^a^	Moderate to high anxiety (DASS), PR (95% CI)	Moderate to high depression (DASS), PR (95% CI)	High work exhaustion, PR (95% CI)	Decreased overall well-being, PR (95% CI)
Age >40 years	0.46 (0.40-0.54)	0.53 (0.46-0.62)	0.49 (0.43-0.56)	0.67 (0.63-0.72)	0.89 (0.86-0.93)
Female	1.16 (0.96-1.40)	1.36 (1.11-1.67)	0.94 (0.81-1.11)	1.18 (1.08-1.28)	1.06 (1.00-1.12)
Underrepresented groups^b^	0.79 (0.62-1.02)	0.99 (0.79-1.24)	0.74 (0.59-0.93)	0.92 (0.83-1.02)	0.91 (0.84-0.98)
Annual household income <$70,000	1.24 (1.06-1.44)	1.43 (1.22-1.67)	1.39 (1.21-1.59)	0.94 (0.87-1.00)	0.97 (0.93-1.02)
Children <18 years old living at home	0.96 (0.83-1.12)	0.85 (0.73-0.99)	0.75 (0.65-0.86)	1.01 (0.94-1.07)	0.98 (0.94-1.03)
High number of stressors^c^	2.17 (1.86-2.54)	2.18 (1.86-2.56)	1.51 (1.32-1.72)	1.37 (1.29-1.46)	1.43 (1.37-1.50)
Staff versus faculty and postdoctoral fellows	0.81 (0.68-0.97)	1.09 (0.89-1.33)	0.94 (0.80-1.11)	0.85 (0.79-0.92)	0.90 (0.85-0.95)
Exposure to coronavirus disease	1.48 (1.19-1.84)	1.37 (1.09-1.73)	1.28 (1.03-1.59)	1.24 (1.13-1.36)	1.04 (0.97-1.12)
Clinical	0.92 (0.76-1.11)	1.21 (1.01-1.45)	0.98 (0.82-1.16)	1.01 (0.93-1.10)	1.18 (1.12-1.24)
Poor supervisor support^d^	1.58 (1.37-1.83)	1.40 (1.21-1.62)	1.69 (1.48-1.92)	1.54 (1.44-1.64)	1.11 (1.07-1.16)

^a^Prevalence ratios (PRs) and 95% CIs were calculated using Poisson regression models.

^b^Underrepresented groups were those identifying as Black/African American, Native American, Hawaiian/Pacific Islander, or Hispanic.

^c^A high number of stressors was defined as a composite stress score >3 (median).

^d^Poor supervisor support was defined as a supervisor support scale score >2 (median).

A comparison of outcomes between faculty and staff working in clinical settings is shown in Table 3 (univariable analyses in Multimedia Appendix 3). Those working in high-risk settings (intensive care unit, emergency room, or performing procedures likely to generate respiratory aerosols) were more likely to report caring for patients with COVID-19 and experiencing an increased workload since COVID-19 restrictions began, had a worse mean score on changes in well-being, and were more likely to report moderate to high stress and depression, high work exhaustion, and burnout. Multivariable analysis of faculty and staff working in clinical operations showed that caring for patients who had COVID-19 was associated with a higher prevalence of stress, anxiety, burnout, and work exhaustion (Table 4). High-risk clinical work (intensive care unit, emergency department, aerosol-generating procedures) showed similar, albeit weaker associations with these outcomes in multivariable analysis (data not shown). There were no statistically significant differences between clinically active staff and faculty for any outcome. Notably, low supervisor support was strongly associated with all mental health and well-being outcomes, and having a high number of family/home stressors was associated with all outcomes except depression.

**Table 3:** Comparison of work factors and outcomes among all clinicians and between high-risk and non–high-risk clinical groups^a^.

Factors and outcomes		Not working in high-risk clinical settings (N=740)	Working in high-risk clinical settings (N=175)	All clinicians (N=915)	*P* value
**Work factors**					
	Contact with outpatients, n (%)	534 (72.2)	77 (44.0)	611 (66.8)	<.001
	Contact with inpatients, n (%)	143 (19.3)	112 (64.0)	255 (27.9)	<.001
	Working in an intensive care unit, n (%)	0 (0.0)	68 (38.9)	68 (7.4)	<.001
	Working in the emergency room, n (%)	0 (0.0)	51 (29.1)	51 (5.6)	<.001
	Performing procedures that create respiratory aerosols, n (%)	0 (0.0)	106 (60.6)	106 (11.6)	<.001
	Caring for patients with COVID-19^b^, n (%)	123 (16.8)	127 (73.8)	250 (27.6)	<.001
	Increased workload since COVID-19 restrictions began, n (%)	279 (38.0)	85 (49.4)	364 (40.2)	.006
	Supervisor support scale (range 1-5), mean (SD)	2.5 (1.1)	2.4 (1.1)	2.5 (1.1)	.50
**Outcomes**					
	Worse overall well-being due to COVID-19–related work or life changes, n (%)	500 (67.9)	127 (73.0)	627 (68.9)	.20
	Worse financial well-being due to COVID-19–related work or life changes, n (%)	313 (42.6)	107 (61.5)	420 (46.2)	<.001
	Worse physical well-being due to COVID-19–related work or life changes, n (%)	339 (46.1)	100 (57.1)	439 (48.2)	.009
	Worse mental well-being due to COVID-19–related work or life changes, n (%)	564 (76.5)	141 (81.0)	705 (77.4)	.20
	Worse social well-being due to COVID-19–related work or life changes, n (%)	629 (85.7)	149 (85.1)	778 (85.6)	.85
	Mean well-being score, mean (SD)	2.2 (0.4)	2.1 (0.5)	2.2 (0.5)	.001
	Moderate to high depression (DASS), n (%)	108 (15.1)	37 (21.6)	145 (16.4)	.04
	Moderate to high anxiety (DASS), n (%)	125 (17.6)	27 (15.8)	152 (17.2)	.58
	Moderate to high stress (DASS), n (%)	93 (13.0)	35 (20.3)	128 (14.5)	.01
	High work exhaustion, n (%)	342 (46.8)	105 (60.7)	447 (49.5)	.001
	High overall burnout, n (%)	233 (32.0)	74 (42.8)	307 (34.0)	.007

^a^The high-risk group reported working in an emergency room, intensive care unit, or performing procedures generating respiratory aerosols. Missing values for each variable (range 0%-3.5%) were omitted from percentage calculations. Percentages may not total 100 due to rounding. Categorical variables are displayed as n (%), while continuous variables are displayed as mean (SD). A chi-square test was used for categorical variables, while a *t* test was used for continuous variables.

^b^COVID-19: coronavirus disease.

**Table 4:** Multivariate associations between personal factors, work factors, and well-being among participants doing clinical work (N=915)^a^.

Variable	Moderate to high stress (DASS), PR (95% CI)	Moderate to high anxiety (DASS), PR (95% CI)	Moderate to high depression (DASS), PR (95% CI)	High overall burnout, PR (95% CI)	High work exhaustion, PR (95% CI)	Decreased overall well-being, PR (95% CI)
Age >40 years	0.56 (0.39-0.81)	0.73 (0.53-1.00)	0.60 (0.43-0.84)	0.77 (0.64-0.93)	0.81 (0.71-0.93)	0.89 (0.82-0.96)
Female	1.26 (0.79-2.00)	1.47 (0.90-2.39)	1.19 (0.77-1.85)	1.18 (0.92-1.51)	1.20 (0.99-1.45)	1.08 (0.97-1.20)
Underrepresented groups^b^	0.56 (0.32-0.98)	0.74 (0.46-1.20)	0.60 (0.35-1.05)	0.66 (0.46-0.94)	0.96 (0.78-1.20)	0.90 (0.78-1.04)
Annual household income <$70,000	1.65 (1.11-2.47)	1.59 (1.11-2.29)	1.46 (1.02-2.11)	1.13 (0.89-1.44)	0.85 (0.72-1.01)	0.91 (0.82-1.01)
Children <18 years old living at home	0.97 (0.68-1.38)	1.07 (0.78-1.47)	0.91 (0.66-1.26)	1.09 (0.90-1.32)	1.06 (0.92-1.21)	0.90 (0.83-0.98)
High number of stressors^c^	1.92 (1.29-2.86)	1.76 (1.22-2.53)	1.23 (0.88-1.70)	1.47 (1.20-1.81)	1.33 (1.15-1.54)	1.27 (1.16-1.39)
Staff	0.97 (0.64-1.46)	1.51 (0.97-2.35)	1.10 (0.74-1.64)	0.88 (0.71-1.10)	1.11 (0.95-1.31)	0.92 (0.84-1.01)
Caring for patients with coronavirus disease	1.73 (1.22-2.46)	1.60 (1.14-2.23)	1.25 (0.88-1.79)	1.38 (1.14-1.67)	1.28 (1.11-1.46)	0.99 (0.91-1.09)
Poor supervisor support^d^	1.93 (1.33-2.81)	1.69 (1.22-2.35)	1.96 (1.39-2.76)	1.99 (1.61-2.47)	1.62 (1.39-1.88)	1.16 (1.06-1.26)

^a^Prevalence ratios (PRs) and 95% CIs were calculated using Poisson multiple regression.

^b^Underrepresented groups were those identifying as Black/African American, Native American, Hawaiian/Pacific Islander, or Hispanic.

^c^A high number of stressors was defined as a composite stress score >3 (median).

^d^Poor supervisor support was defined as a supervisor support scale score >2 (median).

## Discussion

### Principal Results

The EMPOWER study found a high prevalence of stress, anxiety, depression, work exhaustion, burnout, and worsened well-being among clinical and nonclinical university employees surveyed approximately 4 to 5 weeks after work-from-home policies were implemented for those performing work deemed “nonessential” during the crisis phase of the pandemic. These findings uniquely highlight the associations of health and well-being with additional personal and work factors beyond those addressed in existing studies of HCWs during the SARS-CoV-2 pandemic. Importantly, our study also reports on workers outside of clinical medicine, whose health and well-being has been minimally studied. A unique finding of this study is that the factors with the strongest consistent associations with all health and well-being outcomes in both clinical and nonclinical workers were items from the FSSB-SF, a measure of general perception of family-specific supervisory support [9], and a sum of 8 stressors related to family/home life and financial security. Perceived supervisor support for family is a pathway through which employees develop perceptions of organizational support [15], plays a major role influencing the health and well-being of workers [16], and is associated with reduction in work-family conflict, improved well-being, and increased job satisfaction [15,17]. Importantly, family-supportive supervisor behavior can be modified by employer policies and practices.

### Limitations

Limitations of this study include its cross-sectional design, so associations between potential risk factors and outcomes of health and well-being may not be causal. In particular, participants with poorer well-being might differentially report supervisor behaviors. The overall response rate of 34.5% means that the respondents may not be fully representative of all university employees. Faculty were less likely to participate than were staff (19.7% versus 40%), and comparisons between these groups should be interpreted with caution. For instance, faculty were more likely to report increased workload and more work exhaustion since the onset of the pandemic; this difference may be due to differential reporting by faculty, or because faculty were in fact busier and more exhausted and thus less likely to respond. Since the survey was anonymous, our study relies entirely on self-reported data. We studied employees of one university, who may not be representative of other workforces, including lower-paid workers. The St. Louis region was an early adopter of physical distancing and has had a later peak of SARS-CoV-2 and a lower incidence of patients with COVID-19 than some other areas of the United States.

Strengths of the study include its large size, examination of employees who are not in health care, and evaluation of both family/home stressors and workplace factors including supervisor support. To our knowledge, this is the first large US study of multiple mental health and well-being outcomes related to the pandemic outside of a HCW population. We are conducting repeated surveys to track changes in individual health and well-being over time, and to allow more robust causal inferences.

### Comparison With Prior Work

Our findings among clinical workers, both faculty (primarily physicians) and staff (primarily nurses), are broadly consistent with findings from other cross-sectional studies of HCWs caring for patients with COVID-19. A study of 1257 HCWs in China used different instruments and found a higher prevalence of depression and anxiety than seen in our study [2]. Their study reported that HCWs directly involved in the care of patients with COVID-19 were at a greater risk of anxiety and depression, similar to our findings of increased risks of stress, anxiety, burnout, and work exhaustion. A study of 906 HCWs in Singapore and India [18], using the DASS-21, found moderate to severe stress in 3.8%, anxiety in 2.2%, and depression in 8.7%, much lower than the prevalence of 14.5%, 17.2%, and 16.4% seen in our study. Our finding that family/home stressors and supervisor support for family-work balance were strongly associated with mental health and well-being outcomes are consistent with the findings of a recent review of psychological reactions of HCWs during past epidemics [19]. Their analyses showed that responsibilities of caring for family members and lower household income were associated with poorer mental health outcomes among HCWs. Although HCWs caring for patients with COVID-19 had worse mental well-being than their fellow faculty and staff, those working from home or on-site in nonclinical roles also had appreciable rates of poor outcomes. Although we do not have baseline measures for the well-being and mental health outcomes in our study, respondents described altered well-being related to COVID-19–related work/life changes, with 14.6% reporting “much worse” and 68% reporting “much worse” or “somewhat worse” mental well-being. These findings are strikingly similar to those of an April 2020 poll by the Kaiser Family Foundation. Among those who had not experienced job or income loss, 15% reported major negative impacts on their mental health from worry or stress over coronavirus, and 54% reported some negative mental health impacts [20]. Our findings are also supported by results from a recent national online survey conducted among US adults, which compared responses in April 2020 to those from the National Health Interview Survey in 2018 [21]. This study found a higher prevalence of serious psychological distress (13.6% versus 3.9%) in 2020, with younger age and lower income predicting a higher prevalence of distress.

University staff and to some extent faculty are representative of the larger nonclinical workforce that is undergoing uniquely stressful circumstances that blur the boundaries between work and family as people work from home, find it difficult to work because their children’s schools and daycares are closed, or worry about bringing an infection home to their families. Although frontline HCWs are at uniquely high risk due to their work, our study shows that effects of family and home stresses and of supervisor support play a large role in their health and well-being. Appreciation of these factors has been largely missing from studies of risk factors for mental health and well-being among HCWs during this pandemic. These same family and home stresses and supervisor support also influence the health of the broader working population. As the pandemic continues in the months and perhaps years to come, our concern over the mental health and well-being of HCWs must broaden to include other worker groups as well.

There are many possible interventions to address the health and well-being of the clinical and nonclinical workforces. A systematic review found that organizational and social support, clear communication, and having a sense of control were protective factors for adverse mental health outcomes among HCWs during prior epidemics [22]. Recent publications have stressed the importance of robust organizational responses to address the mental health and well-being of frontline HCWs [6,23]. Many of these interventions should be applicable outside of the health care setting. Although interventions aimed at improving resilience among individual workers may lead to improvements in burnout rates and other well-being measures, organizational-level interventions that reduce perceived work demands or increase resources are generally more effective [24]. Our data would suggest that organizations should explicitly focus on improving supervisor support for work-family issues. Evaluation of interventions training supervisors in family-supportive behaviors, including a study in HCWs, have suggested that such training is associated with improved reports of physical health, job satisfaction, job engagement, and decreased intent to leave the current job [25,26]. Future research should include longitudinal studies to follow mental well-being over time, include more workers outside of health care to better understand the effects on the broader population, and test both individual-level and institutional-level interventions to mitigate the effects of the pandemic on mental health.

### Conclusions

Both health care and other workers have encountered worsened mental health and well-being as a result of the SARS-CoV-2 pandemic. Employers, health care systems, and public health agencies should begin interventions to improve mental health and overall well-being among HCWs and the broader workforce. In addition to traditional wellness interventions addressing resilience and mental health issues among individual workers, responses should include support for work/family balance and other organizational changes to improve work conditions for health care and other workers.

## Appendix

Multimedia Appendix 1 Definitions and sources of personal factors, work factors, and well-being variables.

Multimedia Appendix 2 Univariable associations between personal factors, work factors, and well-being among all participants.

Multimedia Appendix 3 Univariable associations between personal factors, work factors, and well-being among participants doing clinical work.

## Abbreviations

COVID-19: coronavirus disease
DASS-21: Depression, Anxiety and Stress Scale - 21 Items
EMPOWER: EMPlOyee Well-Being during Epidemic Response
FSSB-SF: Family Supportive Supervisor Behavior Short-Form
HCW: health care worker
PFI: Professional Fulfillment Index
SARS-CoV-2: severe acute respiratory syndrome coronavirus 2

## References

[1] Lai J, Ma S, Wang Y, Cai Z, Hu J, Wei N, Wu J, Du H, Chen T, Li R, Tan H, Kang L, Yao L, Huang M, Wang H, Wang G, Liu Z, Hu S (2020). Factors Associated With Mental Health Outcomes Among Health Care Workers Exposed to Coronavirus Disease 2019. JAMA Netw Open.

[2] Bettinsoli M, Di RD, Napier J, Moretti L, Bettinsoli P, Delmedico M, Piazzolla A, Moretti B (2020). Psychological Impact and Contextual Factors Associated With Physical and Mental Health Conditions of Italian Healthcare Professionals During the Covid-19 Disease Outbreak. PsyArXiv Preprints.

[3] Wu AW, Connors C, Everly GS (2020). COVID-19: Peer Support and Crisis Communication Strategies to Promote Institutional Resilience. Annals of Internal Medicine.

[4] Ripp J, Peccoralo L, Charney D (2020). Attending to the Emotional Well-Being of the Health Care Workforce in a New York City Health System During the COVID-19 Pandemic. Acad Med.

[5] Shanafelt T, Ripp J, Trockel M (2020). Understanding and Addressing Sources of Anxiety Among Health Care Professionals During the COVID-19 Pandemic. JAMA.

[6] Galea S, Merchant RM, Lurie N (2020). The Mental Health Consequences of COVID-19 and Physical Distancing: The Need for Prevention and Early Intervention. JAMA Intern Med.

[7] Petterson S, Westfall J, Miller B (2020). Projected Deaths of Despair from COVID-19. Well Being Trust.

[8] Kannampallil TG, Goss CW, Evanoff BA, Strickland JR, McAlister RP, Duncan J (2020). Exposure to COVID-19 patients increases physician trainee stress and burnout. PLoS One.

[9] Hammer LB, Ernst Kossek E, Bodner T, Crain T (2013). Measurement development and validation of the Family Supportive Supervisor Behavior Short-Form (FSSB-SF). J Occup Health Psychol.

[10] Lovibond SH, Lovibond PF (1996). Manual for the depression anxiety stress scales.

[11] Trockel M, Bohman B, Lesure E, Hamidi MS, Welle D, Roberts L, Shanafelt T (2018). A Brief Instrument to Assess Both Burnout and Professional Fulfillment in Physicians: Reliability and Validity, Including Correlation with Self-Reported Medical Errors, in a Sample of Resident and Practicing Physicians. Acad Psychiatry.

[12] Serxner S, Kichlu R, Ratelis E (2020). Consumer sentiment during a time of global crisis. Optum Inc.

[13] R Foundation for Statistical Computing R: A language environment for statistical computing.

[14] RStudio Inc RStudio: Integrated Development for R.

[15] Kossek EE, Pichler S, Bodner T, Hammer LB (2011). Workplace social support and work-family conflict: a meta-analysis clarifying the influence of general and work-family-specific supervisor and organizational support. Pers Psychol.

[16] King R, Karuntzos G, Casper L (2012). Work-family balance issues and work-leave policies. Handbook of occupational health and wellness.

[17] Hammer LB, Kossek EE, Yragui NL, Bodner TE, Hanson GC (2009). Development and Validation of a Multidimensional Measure of Family Supportive Supervisor Behaviors (FSSB). J Manage.

[18] Chew NW, Lee GK, Tan BY, Jing M, Goh Y, Ngiam NJ, Yeo LL, Ahmad A, Ahmed Khan F, Napolean Shanmugam G, Sharma AK, Komalkumar R, Meenakshi P, Shah K, Patel B, Chan BP, Sunny S, Chandra B, Ong JJ, Paliwal PR, Wong LY, Sagayanathan R, Chen JT, Ying Ng AY, Teoh HL, Tsivgoulis G, Ho CS, Ho RC, Sharma VK (2020). A multinational, multicentre study on the psychological outcomes and associated physical symptoms amongst healthcare workers during COVID-19 outbreak. Brain Behav Immun.

[19] Kisely S, Warren N, McMahon L, Dalais C, Henry I, Siskind D (2020). Occurrence, prevention, and management of the psychological effects of emerging virus outbreaks on healthcare workers: rapid review and meta-analysis. BMJ.

[20] Kirzinger A, Kearney A, Hamel L, Brodie M (2020). KFF Health Tracking Poll - Early April 2020: The Impact of Coronavirus on Life in America. Kaiser Family Foundation.

[21] McGinty EE, Presskreischer R, Han H, Barry CL (2020). Psychological Distress and Loneliness Reported by US Adults in 2018 and April 2020. JAMA.

[22] De Brier N, Stroobants S, Vandekerckhove P, De Buck E (2020). Factors affecting mental health of health care workers during coronavirus disease outbreaks: a rapid systematic review. PsyArXiv Preprints.

[23] Dzau VJ, Kirch D, Nasca T (2020). Preventing a Parallel Pandemic — A National Strategy to Protect Clinicians’ Well-Being. N Engl J Med.

[24] Panagioti M, Panagopoulou E, Bower P, Lewith G, Kontopantelis E, Chew-Graham C, Dawson S, van MH, Geraghty K, Esmail A (2017). Controlled Interventions to Reduce Burnout in Physicians: A Systematic Review and Meta-analysis. JAMA Intern Med.

[25] Odle-Dusseau HN, Hammer LB, Crain TL, Bodner TE (2016). The influence of family-supportive supervisor training on employee job performance and attitudes: An organizational work-family intervention. J Occup Health Psychol.

[26] Hammer LB, Kossek EE, Anger WK, Bodner T, Zimmerman KL (2011). Clarifying work-family intervention processes: the roles of work-family conflict and family-supportive supervisor behaviors. J Appl Psychol.

