# Correction: Work-Related and Personal Factors Associated With Mental Well-Being During the COVID-19 Response: Survey of Health Care and Other Workers

**DOI:** 10.2196/29069

**Published:** 2021-04-09

**Authors:** Bradley A Evanoff, Jaime R Strickland, Ann Marie Dale, Lisa Hayibor, Emily Page, Jennifer G Duncan, Thomas Kannampallil, Diana L Gray

**Affiliations:** 1 Washington University School of Medicine St. Louis, MO United States; 2 Healthier Workforce Center of the Midwest University of Iowa Iowa City, IA United States; 3 Human Resources Washington University in St. Louis St. Louis, MO United States

In “Work-Related and Personal Factors Associated With Mental Well-Being During the COVID-19 Response: Survey of Health Care and Other Workers” (J Med Internet Res 2020;22(8):e21366) the authors noted two errors in the reference list.

In the originally published paper, the following reference was omitted from the reference list:

Wu AW, Connors C, Everly GS. COVID-19: Peer Support and Crisis Communication Strategies to Promote Institutional Resilience. Annals of Internal Medicine 2020 Jun 16;172(12):822-823. [doi: 10.7326/m20-1236]

This citation has been inserted as Reference 3 in the corrected version of the paper.

In addition, References 20 and 21 were duplicate references in the originally published paper. In the corrected version, this reference (McGinty et al) remains as Reference 21. The remaining references have been renumbered accordingly to adjust for these changes. The corrected and renumbered list of references appears below and in the corrected paper.

The correction will appear in the online version of the paper on the JMIR Publications website on April 9, 2021, together with the publication of this correction notice. Because this was made after submission to PubMed, PubMed Central, and other full-text repositories, the corrected article has also been resubmitted to those repositories.
