# Supplementary material for: Work-Related and Personal Factors Associated With Mental Well-Being During the COVID-19 Response: Survey of Health Care and Other Workers
Source: J Med Internet Res. 2020 Aug 25;22(8):e21366. doi: 10.2196/21366 (PMC7470175; doi:10.2196/21366)
Supplement: Multimedia Appendix 1 [file jmir_v22i8e21366_app1.docx]

Supplementary Table 1: Definitions and sources of personal factors, work factors and wellbeing variables

| Variable | Definition/Source |
| --- | --- |
| Under-represented groups | American Indian/Alaskan Native or Black/African American or Native Hawaiian/Other Pacific Islander or Hispanic/Latin origin |
| Stressed about childcare | “Somewhat”, “Quite a bit” or “Extremely” stressed about childcare |
| Stressed about home schooling | “Somewhat”, “Quite a bit” or “Extremely” stressed about home schooling |
| Stressed about relatives | “Somewhat”, “Quite a bit” or “Extremely” stressed about care for relatives |
| Stressed about essential supplies | “Somewhat”, “Quite a bit” or “Extremely” stressed about access to essential supplies |
| Stressed about being infected | “Somewhat”, “Quite a bit” or “Extremely” stressed about being infected with COVID-19 |
| Stressed about friends/family getting infected | “Somewhat”, “Quite a bit” or “Extremely” stressed about friends or family being infected with COVID-19 |
| Stressed about keeping job | “Somewhat”, “Quite a bit” or “Extremely” stressed about keeping job |
| Stressed about personal finances | “Somewhat”, “Quite a bit” or “Extremely” stressed about personal finances |
| Number of stressors | Count of reported stressors (childcare, home schooling, relatives, essential supplies, being infected with COVID-19, friends or family being infected with COVID-19, keeping job and personal finances) |
| Any exposure to COVID-19 | Positive COVID-19 test/medical diagnosis or exposure to someone with a COVID-19 positive test/medical diagnosis or member of household diagnosed with COVID-19 |
| High risk clinical work (clinical workers only) | Working in an Intensive Care Unit/Working in the Emergency Room/ Performing procedures that create respiratory aerosol |
| Caring for COVID19 patients (clinical workers only) | Answered ‘Yes” to “In your current clinical role, are you seeing or caring for patients that have tested positive for Covid-19?” |
| Supervisor support scale | Mean scale of supervisor support questions from Family Supporting Supervisor Behavior (Short-Form) |
| Increased workload since COVID-19 restrictions began | Workload has “increased a lot” or “increased a little” since the COVID-19 restrictions began. |
| Worse overall wellbeing due to COVID-19 related work/life changes | Answered “much worse” or “somewhat worse” to “To what extent have COVID-19-related work/life changes impacted your overall well-being” from OPTUM survey |

Supplementary Table 1: Definitions and sources of personal factors, work factors and wellbeing variables (continued)

| Variable | Definition/Source |
| --- | --- |
| Worse financial wellbeing due to COVID-19 related work/life changes | Answered “much worse” or “somewhat worse” to “To what extent have COVID-19-related work/life changes impacted your financial well-being” from OPTUM survey |
| Worse physical wellbeing due to COVID-19 related work/life changes | Answered “much worse” or “somewhat worse” to “To what extent have COVID-19-related work/life changes impacted your physical well-being” from OPTUM survey |
| Worse mental wellbeing due to COVID-19 related work/life changes | Answered “much worse” or “somewhat worse” to “To what extent have COVID-19-related work/life changes impacted your mental well-being” from OPTUM survey |
| Worse social wellbeing due to COVID-19 related work/life changes | Answered “much worse” or “somewhat worse” to “To what extent have COVID-19-related work/life changes impacted your social well-being” from OPTUM survey |
| Mean wellbeing score | Computed mean score of overall, financial, physical, mental and social well-being items |
| Moderate to high depression (DASS) | DASS 21 Depression component “Moderate” “Severe” or “Extremely Severe” |
| Moderate to high anxiety (DASS) | DASS 21 Anxiety component “Moderate” “Severe” or “Extremely Severe” |
| Moderate to high stress (DASS) | DASS 21 Stress component “Moderate” “Severe” or “Extremely Severe” |
| High work exhaustion | Work exhaustion score > 1.33 from Professional Fulfillment index (PFI) |
| High interpersonal disengagement (clinical workers only) | Interpersonal disengagement score > 1.33 from Professional Fulfillment index (PFI) |
| High overall burnout | Overall burnout score > 1.33 from Professional Fulfillment index (PFI) |
