# Supplementary material for: Work-Related and Personal Factors Associated With Mental Well-Being During the COVID-19 Response: Survey of Health Care and Other Workers
Source: J Med Internet Res. 2020 Aug 25;22(8):e21366. doi: 10.2196/21366 (PMC7470175; doi:10.2196/21366)
Supplement: Multimedia Appendix 2 [file jmir_v22i8e21366_app2.docx]

Supplementary Table 2. Univariable associations between personal factors, work factors, and well-being among all participants (n = 5550, Prevalence Ratio (PR) calculated using Poisson regression models).

| Variable | *Moderate to high stress (DASS)* | | | *Moderate to high anxiety (DASS)* | | | *Moderate to high depression (DASS)* | | *High work exhaustion* | | *Decreased overall wellbeing* | |
| --- | --- | --- | --- | --- | --- | --- | --- | --- | --- | --- | --- | --- |
|  | | PR | CI | | PR | CI | PR | CI | PR | CI | PR | CI |
| Age above 40 years | | **0.44** | **(0.38 - 0.51)** | | 0.49 | **(0.43 - 0.57)** | **0.47** | **(0.42 - 0.54)** | **0.68** | **(0.64 - 0.72)** | **0.89** | **(0.86 - 0.93)** |
| Female | | **1.30** | **(1.08 - 1.56)** | | **1.58** | **(1.29 - 1.94)** | 1.03 | (0.88 - 1.20) | **1.19** | **(1.09 - 1.29)** | **1.06** | **(1.01 - 1.12)** |
| Under-represented groups^a^ | | 0.96 | (0.76 - 1.22) | | **1.26** | **(1.03 - 1.54)** | 0.90 | (0.73 - 1.11) | 0.95 | (0.86 - 1.06) | 0.95 | (0.88 - 1.02) |
| Annual Household Income $70,000 and below | | **1.36** | **(1.18 - 1.56)** | | **1.64** | **(1.42 - 1.88)** | **1.56** | **(1.38 - 1.76)** | 0.97 | (0.91 - 1.04) | 0.97 | (0.93 - 1.02) |
| Children under 18 years old living at home | | 1.13 | (0.98 - 1.29) | | 0.95 | (0.82 - 1.09) | **0.79** | **(0.70 - 0.90)** | **1.12** | **(1.06 - 1.19)** | **1.09** | **(1.05 - 1.14)** |
| High number of stressors^b^ | | **2.33** | **(2.01 - 2.70)** | | **2.39** | **(2.06 - 2.77)** | **1.53** | **(1.35 - 1.73)** | **1.42** | **(1.34 - 1.52)** | **1.44** | **(1.38 - 1.51)** |
| Staff | | 0.91 | (0.77 - 1.08) | | **1.24** | **(1.02 - 1.49)** | 0.95 | (0.81 - 1.10) | **0.83** | **(0.77 - 0.89)** | **0.91** | **(0.86 - 0.95)** |
| Exposure to COVID19 | | **1.58** | **(1.28 - 1.94)** | | **1.49** | **(1.21 - 1.85)** | **1.29** | **(1.05 - 1.58)** | **1.37** | **(1.25 - 1.50)** | **1.17** | **(1.10 - 1.25)** |
| Clinical | | 1.13 | (0.95 - 1.35) | | **1.41** | **(1.20 - 1.67)** | 1.03 | (0.88 - 1.22) | **1.19** | **(1.11 - 1.28)** | **1.24** | **(1.19 - 1.30)** |
| Poor supervisor support^c^ | | **1.64** | **(1.42 - 1.89)** | | **1.44** | **(1.25 - 1.66)** | **1.66** | **(1.46 - 1.89)** | **1.6** | **(1.51 - 1.71)** | **1.16** | **(1.12 - 1.21)** |

^a^Under-represented groups were those identifying as Black/African American, Native American, Hawaiian/Pacific Islander or Hispanic

^b^High number of stressors defined as composite stress score >3 (median)

^c^Poor supervisor support defined as supervisor support scale >2 (median)
