# Supplementary material for: Work-Related and Personal Factors Associated With Mental Well-Being During the COVID-19 Response: Survey of Health Care and Other Workers
Source: J Med Internet Res. 2020 Aug 25;22(8):e21366. doi: 10.2196/21366 (PMC7470175; doi:10.2196/21366)
Supplement: Multimedia Appendix 3 [file jmir_v22i8e21366_app3.docx]

Supplementary Table 3. Univariable associations between personal factors, work factors, and well-being among participants doing clinical work (n = 915, Prevalence Ratio (PR) calculated using Poisson multiple regression).

| Variable | *Moderate to high stress (DASS)* | | *Moderate to high anxiety (DASS)* | | *Moderate to high depression (DASS)* | | *High overall burnout* | | *High work exhaustion* | | *Decreased overall wellbeing* | |
| --- | --- | --- | --- | --- | --- | --- | --- | --- | --- | --- | --- | --- |
|  | PR | CI | PR | CI | PR | CI | PR | CI | PR | CI | PR | CI |
| Age above 40 years | **0.53** | **(0.38 - 0.73)** | **0.64** | **(0.48 - 0.85)** | **0.58** | **(0.43 - 0.78)** | **0.75** | **(0.63 - 0.90)** | **0.80** | **(0.71 - 0.91)** | **0.91** | **(0.84 - 0.98)** |
| Female | 1.40 | (0.88 - 2.22) | **2.02** | **(1.23 - 3.30)** | 1.25 | (0.82 - 1.88) | 1.16 | (0.91 - 1.49) | **1.25** | **(1.03 - 1.51)** | 1.07 | (0.97 - 1.19) |
| Under-represented groups^a^ | 0.95 | (0.57 - 1.58) | 1.22 | (0.81 - 1.84) | 0.91 | (0.56 - 1.49) | 0.82 | (0.60 - 1.13) | 1.00 | (0.81 - 1.23) | 0.91 | (0.80 - 1.05) |
| Annual Household Income $70,000 and below | **1.55** | **(1.11 - 2.17)** | **1.87** | **(1.39 - 2.51)** | **1.52** | **(1.12 - 2.07)** | 1.07 | (0.87 - 1.31) | 0.94 | (0.81 - 1.10) | 0.94 | (0.85 - 1.03) |
| Children under 18 years old living at home | 1.18 | (0.85 - 1.62) | 1.10 | (0.82 - 1.47) | 1.00 | (0.74 - 1.34) | **1.24** | **(1.03 - 1.49)** | **1.17** | **(1.03 - 1.33)** | 0.99 | (0.92 - 1.07) |
| High number of stressors^b^ | **2.39** | **(1.65 - 3.44)** | **2.23** | **(1.61 - 3.09)** | **1.42** | **(1.05 - 1.94)** | **1.63** | **(1.35 - 1.98)** | **1.51** | **(1.31 - 1.73)** | **1.26** | **(1.16 - 1.37)** |
| Staff | 1.15 | (0.81 - 1.64) | **1.88** | **(1.31 - 2.71)** | 1.28 | (0.92 - 1.78) | 0.94 | (0.78 - 1.14) | 1.10 | (0.95 - 1.27) | 0.96 | (0.89 - 1.05) |
| Caring for COVID19 patients | **1.57** | **(1.13 - 2.18)** | **1.36** | **(1.01 - 1.84)** | 1.17 | (0.85 - 1.61) | **1.36** | **(1.13 - 1.64)** | **1.24** | **(1.08 - 1.42)** | 1.01 | (0.93 - 1.10) |
| Poor supervisor support^c^ | **1.97** | **(1.37 - 2.82)** | **1.61** | **(1.18 - 2.21)** | **1.93** | **(1.38 - 2.69)** | **2.01** | **(1.63 - 2.49)** | **1.64** | **(1.41 - 1.90)** | **1.20** | **(1.10 - 1.30)** |

^a^Under-represented groups were those identifying as Black/African American, Native American, Hawaiian/Pacific Islander or Hispanic

^b^High number of stressors defined as composite stress score >3 (median)

^c^Poor supervisor support defined as supervisor support scale >2 (median)
